# Iron-fortified lentils to improve iron (Fe) status among adolescent girls in Bangladesh - study protocol for a double-blind community-based randomized controlled trial

**DOI:** 10.1186/s13063-019-3309-4

**Published:** 2019-05-02

**Authors:** Fakir Md Yunus, Chowdhury Jalal, Kaosar Afsana, Rajib Podder, Albert Vandenberg, Diane M. DellaValle

**Affiliations:** 1 College of Pharmacy and Nutrition, University of Saskatchewan, 104 Clinic Place, Saskatoon, Saskatchewan S7N 2Z4 Canada; 2 Nutrition International, 180 Elgin Street, Suite 1000, Ottawa, Ontario K2P 2K3 Canada; 3 BRAC Health, Nutrition, and Population Program, 75 Mohakhali, BRAC Centre, Dhaka, 1212 Bangladesh; 4 College of Agriculture and Bio-resources, The University of Saskatchewan, Agriculture Building 51 Campus Drive, Saskatoon, Saskatchewan S7N 5A8 Canada; 5 Nutrition, Athletic Training and Exercise Science, Marywood University, 2300 Adams Avenue, Scranton, PA 18509 USA

**Keywords:** Micronutrient deficiency, Iron (Fe), Food-based approach, Food technology, Fortification

## Abstract

**Background:**

Lentils are generally considered to be a nutrient-dense food, and a good source of iron (Fe). This study aims to establish novel evidence of the effectiveness of the consumption of Fe-fortified lentils in improving the body Fe status and thus cognitive performance in non-pregnant adolescent girls in rural Bangladesh, compared to consumption of ordinary lentils.

**Methods:**

We have designed a double-blind (both trial participants and outcome assessors), community-based, cluster-randomized controlled trial among 1260 Bangladeshi adolescent girls between the ages of 10–17 years who are non-smoking, not married, not pregnant, not breastfeeding, and generally healthy at the time of enrollment. The intervention will include three arms who will receive: (1) Fe-fortified lentils; (2) unfortified lentils; or (3) usual intake. Participants will be served a thick preparation of cooked Fe-fortified lentils (37.5 g raw lentils, approximately 200 g cooked lentils) 5 days per week for 85 feeding days (around 4 months) using a locally acceptable recipe. Lentils were fortified with Fe in the laboratory at the Department of Plant Sciences at the University of Saskatchewan in Canada. A subsample of participants (*n* = 360) will be randomly invited to be included in cognitive testing.

**Discussion:**

Data on socio-demographic characteristics, household food security status, adolescent food habits and cognitive testing will be collected at baseline and endline (4 months). Venous blood samples will be collected at baseline, midline (2 months) and endline to measure adolescents’ Fe status. Computerized cognitive testing will include five common measures of attentional (three of attention) and mnemonic functioning (two of memory) carried out using DMDX software. The results of this study will be used to garner support for and to substantiate large-scale production and market expansion of Fe-fortified lentils, and will contribute to knowledge about how to enhance Fe status in adolescents worldwide in resource-poor settings, using staple food crops.

**Trial registration:**

ClinicalTrials.gov NCT03516734. Registered on 24 May 2018.

**Electronic supplementary material:**

The online version of this article (10.1186/s13063-019-3309-4) contains supplementary material, which is available to authorized users.

## Background

Based on data from 2011 to 2012, it was estimated that 26% of non-pregnant non-lactating (NPNL) women and 17.1% of adolescents aged 12–14 years were anemic (< 12.0 g/dl) in Bangladesh, and that prevalence was higher in rural areas [1]. National levels of the reported prevalence of iron deficiency anemia in children aged 6–11 years (hemoglobin < 11.5 g/dl and ferritin < 15.0 μg/L) was 1.3% and in adolescents aged 12–14 years (hemoglobin < 12.0 g/dL and ferritin < 15.0 μg/L) it was 1.8% [2]. Adolescents girls are particularly vulnerable to iron (Fe) deficiency with and without anemia due to their significant growth and development, lifestyle and food habits, and their regular menstrual losses of Fe [3, 4].

Improving dietary Fe intake via Fe supplementation or high-Fe foods is extremely important among adolescent girls living in Bangladesh and other resource-poor settings, in order to maintain nutrition status and growth. Food-based approaches such as Fe fortification have the potential to be sustainable in meeting the increasing demand for Fe among adolescent girls, whereas direct Fe supplementation is often a problem due to poor compliance [5, 6]. Lentils (locally known as *masur dal* in Bangladesh) are a nutrient-dense staple in South Asia, and a good source of Fe. There has been great progress made in lentil fortification and biofortification research over the past decade [7–9]. Lentils grown in Saskatchewan, Canada are rich in Fe (73–90 mg/kg), zinc (Zn, 44–54 mg/kg), and selenium (425–673 μg/kg) [7, 10]. Further research has shown that Saskatchewan-grown lentils are also naturally lower in phytic acid (2.5–4.4 mg/g), indicating that Fe and Zn would be more bioavailable [7, 10–12]. Furthermore, Fe status is associated with improved cognitive performance [13, 14]. Therefore, there is a tremendous opportunity to include lentils as part of a whole-food-based, sustainable solution to the global micronutrient deficiency problem. This community-based trial is designed to examine the effectiveness of Saskatchewan-grown Fe-fortified lentils in improving the Fe status and cognitive performance of adolescent girls in Bangladesh.

## Trial objectives

The primary objective of the study is to determine the effectiveness of an Fe-fortified lentil-based dietary intervention compared to ordinary lentils and usual intake group in improving the Fe status of non-pregnant adolescent girls in Bangladesh. The primary comparison would be Fe-fortified lentils versus non-Fe-fortified lentils and the secondary comparison would be Fe-fortified lentils versus usual intake. We hypothesize that the supplemental food-based Fe from the fortified lentils will improve body Fe status in adolescent girls, and thus cognitive performance, compared to ordinary lentils and usual intake. Furthermore, we will examine the effect of Fe-fortified and non-Fe-fortified Canadian lentil consumption (intervention) on growth (height, body weight, triceps skinfolds, mid-upper arm circumference) of non-pregnant adolescent girls in Bangladesh.

## Material and methods

### Feasibility study

Before designing the effectiveness trial, a feasibility study was conducted in Bangladesh in 2017 to examine the logistics and feasibility of the implementation of a human food intake trial of Fe-fortified lentils among adolescent girls (10–17 years) for 12 weeks [15]. The purpose of the study was to determine the viability of the proposed effectiveness trial. Both thin and thick traditional lentil dishes were prepared based on uncooked/raw lentil amounts of 25 g, 37.5 g, and 50 g/person [15]. The study findings suggested that adolescents were keen to eat cooked lentils over the 3-month study period, and the drop-out rate was 0% (with 5.2% of meal missed by participants). Adolescents’ hunger, fullness, and gastrointestinal discomfort before and after consuming cooked lentil portions were assessed using a visual analog scale (VAS). Higher palatability was observed for the thick preparation of cooked lentils compared to the thin preparation for all three intervention raw amounts of 25 g vs 37.5 g vs 50 g (cooked amount ~ 157.9 g vs 202.7 g vs 256.6 g, respectively). There were no significant differences between uneaten amounts of the lentil dishes containing 37.5 g vs 50 g raw lentils (cooked amount ~ 202.7 g vs 256.6 g, respectively). Moreover, 37.5 g raw amount would provide approximately 86.3% and 46% of the recommended dietary allowance (RDA) for Fe for adolescent girls aged 9–13 and 14–18 years, respectively [15]. Considering the cultural appropriateness of the amount of cooked lentil (*dal*) consumed and its Fe content, the study adopted 37.5 g raw lentil (~ 200 g cooked amount) as the intervention portion size for the future human effectiveness trial.

### Study design

This manuscript describes the protocol for a double-blind (both the trial participants and the outcome assessors), community-based, cluster-randomized controlled trial designed to test the effectiveness of consuming Fe-fortified lentils to improve Fe stores and cognitive performance (attention, and memory) in adolescent girls in rural Bangladesh. Given the context of this problem, the cluster-randomized controlled trial allows us to (1) avoid confounding and ensure representativeness at baseline; (2) minimize risk of contamination between treatment arms; and (3) examine the temporal relationship between our intervention and the outcome.

### Study settings and target population

The effectiveness study will be conducted at the BRAC Adolescent Clubs [16]. These clubs target all adolescent boys and girls in the community, regardless of their school attendance, marital status, or socio-economic status. The clubs provide a unique opportunity for adolescents to socialize in both rural and urban settings. Each club has a membership of 25–40 adolescent boys and girls aged 10–19 years. The clubs operate in the afternoons in BRAC school environments. In the absence of such facilities, a room is rented locally by BRAC. The clubs have various activities to encourage the participation of adolescent girls and boys such as life-skill-based education (LSBE) sessions, access to mini-library facilities, cultural activities, and sports. BRAC provides all materials related to these activities, e.g., books, magazines, and games equipment. The clubs will be selected from four subdistricts (*Upazilas*) i.e., Muktagacha, Nanadail or Mymensingh Sadar (Central), Bhaluka, and Gaffargaon, of the Mymensingh district.

**Fig. 1:**
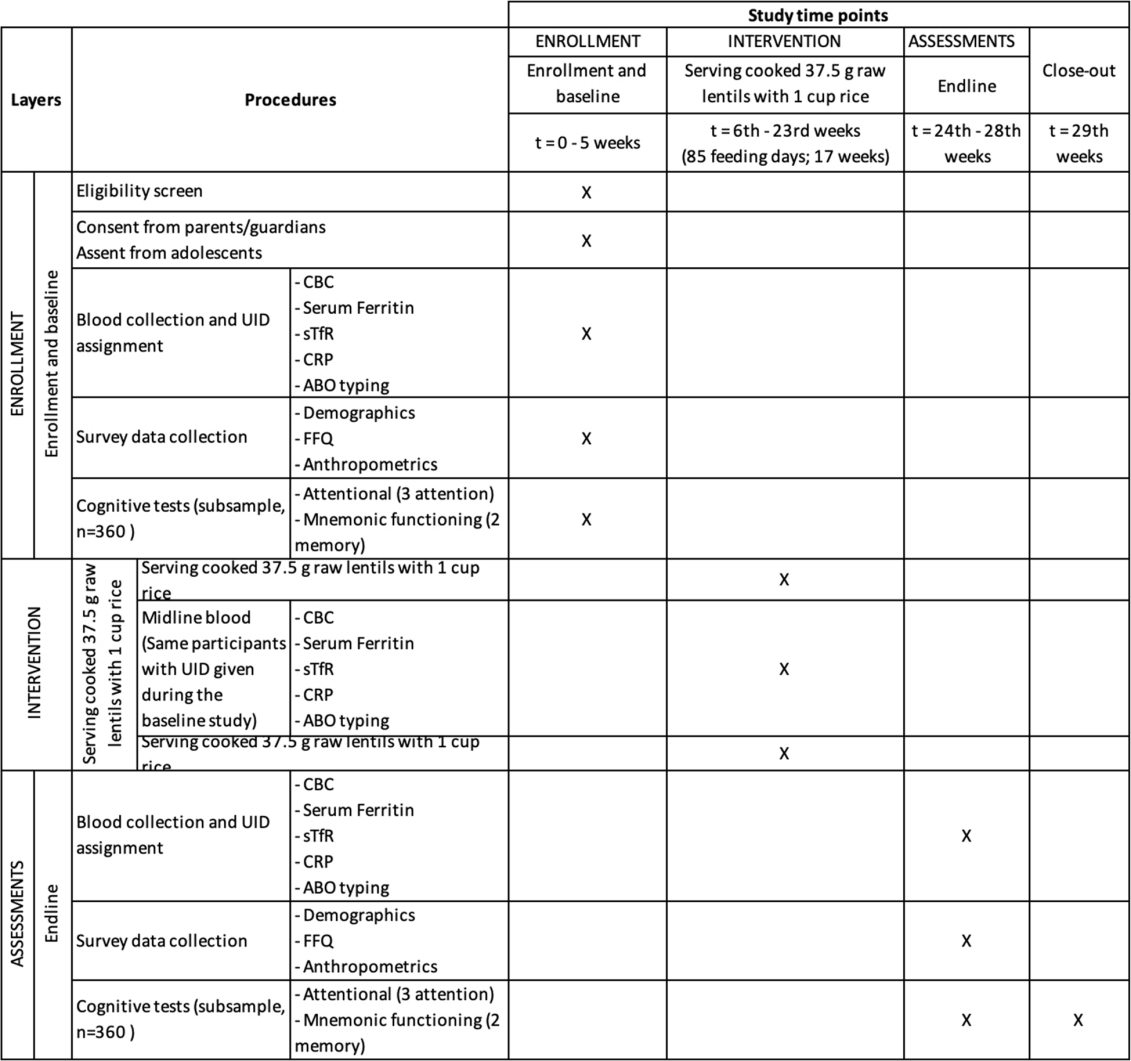
Flow chart of iron-fortified lentil feeding trial

The study has been carefully designed to ensure that all of the adolescent girls between the ages of 10–17 years meet the study inclusion criteria: non-smoking, not married, not pregnant, not breastfeeding, and generally healthy. Adolescent girls who choose not to participate, are ill during recruitment, or are known to have infectious disease will be excluded. Standard Protocol Items: Recommendations for Intervention Trials (SPIRIT) 2013 (Fig. 1) will be used to report the study [17]. Table 1 presents a summary of the trial according to the World Health Organization (WHO) Trial Registration Minimal Data Set as described by Moja et al. [18].

**Table 1:** Summary of the trial according to the World Health Organization (WHO) trial registration minimal data set

	Data	Information
1	Trial identification number (unique trial number)	ClinicalTrials.gov NCT03516734
2	Trial registration date	24 May 2018
3	Secondary IDs	Bio# 17–177 (ERC, University of Saskatchewan) IRB#1139116–2 (IRB, Marywood University) BMRC/NREC/2016–2019/455 (Bangladesh Medical and Research Council)
4	Funding source(s)	Global Institute for Food Security, University of Saskatchewan, Canada; Nutrition International, Canada
5	Primary Sponsor	Albert Vandenberg, PhD (University of Saskatchewan)
6	Secondary Sponsor	Carol J Henry, PhD (University of Saskatchewan)
7	Responsible contact person (contact for public queries)	Carol J Henry, PhD, College of Pharmacy and Nutrition, University of Saskatchewan, 104 Clinic Place, Saskatoon, SK, S7N 2Z4, Saskatchewan, Canada. carol.henry@usask.ca
8	Research contact person (contact for scientific queries)	Diane M DellaValle, PhD, RDN, LDN, Nutrition, Athletic Training and Exercise Science, Department of Nutrition and Dietetics, Marywood University, 2300 Adams Avenue Scranton, PA 18509, USA. ddellavalle@marywood.edu
9	Title of the study (brief title)	Iron-fortified lentils to improve iron (Fe) status among adolescent girls in Bangladesh
10	Official scientific title of the study	Iron-fortified lentils to improve iron (Fe) status among adolescent girls in Bangladesh - study protocol for a double-blind randomized controlled trial.
11	Research ethics review and country of recruitment	• Ethical approved received from 1. University of Saskatchewan REB (Ref: Bio# 17–177) 2. Marywood University IRB (Ref: IRB#1139116–2) 3. Bangladesh Medical and Research Council. (Ref: BMRC/NREC/2016–2019/455) • Country of recruitment: Bangladesh
12	Health condition studied	Iron deficiency
13	Intervention	Fe-fortified lentils
14	Key inclusion and exclusion criteria	Inclusion criteria: adolescent girls aged 10–17 years, non-smoking, not pregnant, not breastfeeding, and generally healthy Exclusion criteria: adolescent girls with active illness during recruitment, or with known infectious disease
15	Study type	Double-blind, community-based, cluster-randomized controlled trial
16	Trial start date (anticipated)	Mid-September 2018
17	Target sample size (total)	1260 adolescent girls.
18	Recruitment status	Not yet recruiting
19	Primary outcome	Serum ferritin level and cognitive performance of the adolescent girls
20	Key secondary outcomes	1. Growth (height, body weight, triceps skinfolds, mid-upper arm circumference) of non-pregnant adolescent girls in Bangladesh 2. Blood hemoglobin level of non-pregnant adolescent girls in Bangladesh

### Lentil fortification with Fe

Lentils were fortified with Fe at the Saskatchewan Food Industry Development Center Inc. (Food Center) in collaboration with the Department of Plant Sciences of the Crop Development Center (CDC) of The University of Saskatchewan, Canada. A small red lentil variety (CDC Maxim) was used, similarly to the previously developed protocol [19]. In brief, the fortification method was developed to identify the most suitable product types (decorticated unsplit lentil dal), appropriate methods for fortification, dosage and colorimetric changes, and the storability of Fe-fortified lentil dal among three different Fe fortifiers (FeSO_4_·7H_2_O, NaFeEDTA and FeSO_4_·H_2_O). NaFeEDTA was found to be the most suitable Fe fortifier of lentil dal at 1600 μg g^− 1^ (fortifier Fe concentration), providing 13–14 mg more Fe 100^− 1^ g of dal [19]. The fortified lentils were bagged in 20-kg airtight polyvinyl bags and stored at room temperature before being shipped to Bangladesh.

### Intervention

There will be three arms in this effectiveness trial. Arm 1 will receive Fe-fortified lentils, arm 2 will receive unfortified lentils, and arm 3 will receive no intervention i.e. no additional lentil (usual intake group) and will serve as a control group. The usual intake group will serve as control for the lentil-related research questions, but not those pertaining to the fortification itself. Participants will not be asked to change anything about their food intake during the study, including lentil intake. Participating adolescents will be served a thick preparation of cooked lentils (37.5 g raw lentil) 5 days per week for 85 feeding days (around 4 months).

A team of 3 will manage the distribution of the lentil preparation: (1) a locally-recruited cook, (2) a research assistant who will measure and serve the cooked lentils, and (3) an Adolescent Club leader who will assist in serving and ensuring that safe drinking water is available. A locally acceptable, standard lentil dal recipe identified during the earlier feasibility study will be used. The recipe for thick cooked lentils will include turmeric 5 g, chopped onion 40 g, garlic 8 g, green chili 3 g, water 700 ml, salt 1.5 teaspoon, soybean oil 10 teaspoons, and one small bay leaf (*tejpata*) per 100 g uncooked lentils. The average cooking time would be around 18 min, and the approximate weight (after cooking of 37.5 g raw lentil) would be approximately 200 g [15]. All adolescent boys and girls who attend the BRAC Adolescent Clubs will be offered the cooked lentils during the period of the intervention; however, the information on those girls not meeting the inclusion criteria will not be analyzed.

### Sample size, randomization and blinding

Considering the lower estimation of the expected difference in mean serum ferritin (sFer) (5 ±20 μg/L) with 80% power at *p* < 0.05 significance level and inter-cluster correlation (ICC) at 0.025, a total of 48 clusters will be selected falling under 16 blocks. Each block will have 3 clubs resulting in a total of 48 clubs. Each club is considered as cluster. Within each cluster, 27 eligible adolescent girls will be selected. Clusters will be randomly assigned to the intervention within each block. In this cluster RCT, units of randomization will be Adolescent Clubs where data will be collected from individual girls. First, a total of 48 clubs will be randomly selected out of 75 clubs. Each cluster will be randomly assigned under 16 blocks. There will be three clusters in each block and three study arms (Fe-fortified lentil, non-Fe-fortified lentil, or usual intake) will be then randomly allocated within blocks using computer-generated random assignments. Equal numbers of clubs (*n* = 16) will be assigned to each arm and there will be equal numbers of participants (*n* = 420) in each arm. Finally, 420 adolescent girls will be included in each arm including an additional 20% to account for loss to follow up. The total sample size including all three arms will be 1260 adolescent girls. For the cognitive testing, a subsample of the intervention group (*n* = 80 adolescent girls in each group, 5 per cluster, a total of *n* = 240 participants) will be selected assuming a two-tailed, 5% type I error rate with 80% power, and an ICC of 0.025. This is based on a recent biofortified iron study with 70% effect size [13, 20]. We further increased the sample size to a total of 360 adolescent girls (e.g., *n* = 120 adolescent girls in each group, considering a 50% attrition rate). We assumed a 20% attrition rate in our first outcome; however, we assume the attrition rate may be higher in the cognitive part as it would take longer, plus many of the participants may have not seen or physically touched a laptop in their lifetime. We assume these will create a greater burden to them resulting in a higher attrition rate. Cluster numbers were sequential at 4-digit point because we wanted to make our UID an 8-digit number: 2 digits for the club ID, 4 digits for cluster, and 2 digits for the participant. This makes the UID more unique and will avoid confusion among the research assistants about each part of the UID. A potential problem with sequential numbering (e.g., 1 .. 16) would be that the different digits used for the participants may lead to different sizes of the UID stickers, and thus to difficulty using the UID stickers during blood sample collection (e.g., on paperwork, blood sample tubes, etc.).

The study will follow the double-blind strategy in distributing the Fe-fortified or non-Fe-fortified lentils to the participants. The double-blind strategy (blinding both of trial participants and outcome assessors) will be carried out by a third party under the direct supervision of the principal investigator of the project. Each lentil packet (stiff cardboard box) will contain double-layered color-coded bags. The outer layer will be marked with an adhesive colored sticker. The inner layer (food-grade bag) will be representing Fe-fortified or non-Fe-fortified lentils. Color-coded packets will be delivered to the intervention Adolescent Clubs. Blinding will be broken within 24 h availability in any instance of unexpected or untoward conditions, for example, mass hysteria, mass diarrhea linked to served lentils, unexpected death, or life-threatening condition of the participants, etc. During the data collection period, the researchers will be in touch with the local research assistants on a daily basis to receive daily updates on the research from the field. If required, an urgent message will be sent by field researchers to all the study investigators if there are any unexpected or untoward conditions. All the Institutional Review Boards (IRBs) will be informed if such a situation occurs.

### Data collection tools and technique

**Fig. 2:**
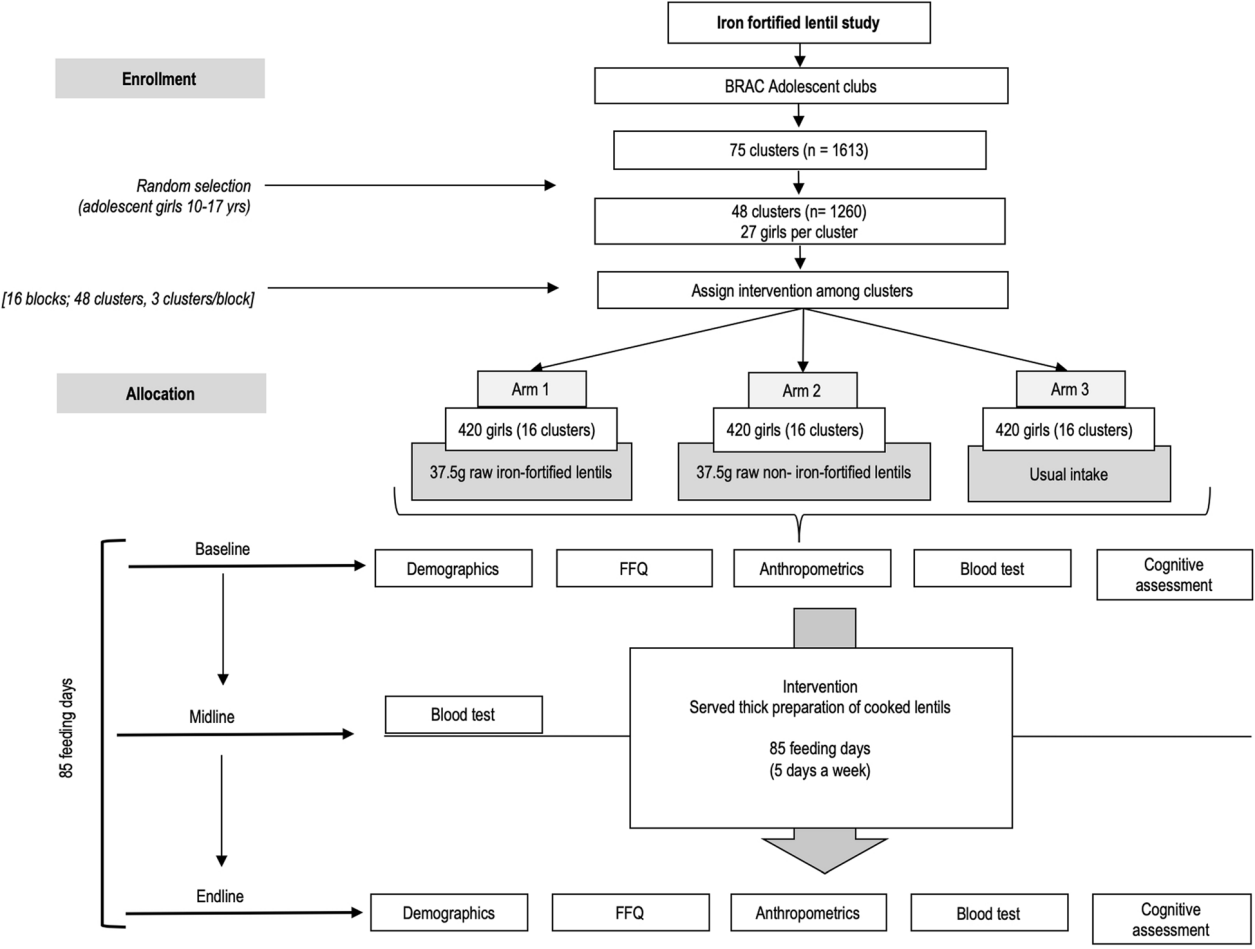
Flow chart for the 85-day iron-fortified lentil feeding trial. Demographics, food frequency questionnaire (FFQ), anthropometrics, blood test, cognitive assessment

Four types of data will be collected in three rounds. These include survey data, and data on cognitive performance, venous blood samples, and daily lentil consumption. Round 1 (baseline) will include all forms of data. Round 2 (midline) at 2 months will only include blood sample data. Round 3 (end of study) at 4 months will include the same data as at baseline (Fig. 2).

First of all, healthy female adolescents aged 10–17 years, who are non-smoking, not pregnant, and not breastfeeding, and willing to participate will be included in the study. Those who give consent will be invited to attend the BRAC Adolescent Clubs and venous blood samples will be collected in a private setting within the club by a 3-person team consisting of (1) a medical technologist, (2) a female research assistant and (3) a male research assistant. BRAC local staff will be present to monitor the entire process. Albendazole (400 mg in tablet form) will be provided so that participants are dewormed at the time of the feeding trial meaning that they are free from parasites, such as roundworm, flukes, and tapeworm infestation. Earlier studies suggest that worm infestation is linked with anemia and deworming reduces anemia among children [31–33].

All participants will be provided with the contact details of the study coordinator to provide guidance on future questions and concerns. A copy of the consent form will be given to the participants. Publications and scientific presentations of the findings from the study will be presented in aggregate, and the identity of individual participants will be kept confidential. The study will use study codes for hard-copy data documents (e.g., completed questionnaire) instead of recording identifying information and we will maintain a separate document that links the study code to the subjects’ identification information. This document will be locked up in a separate location with restricted access to it (e.g., only allowing access for primary investigators). Laptop computers used by researchers that collect and manage the data will be protected with whole-drive disk encryption that prevents data access if the laptop is lost or stolen. Face sheets containing identifiers (e.g., names and addresses) from survey instruments containing data will be removed after receiving them from study participants. Sensitive identifiers (e.g., names and addresses) will not be permitted to be stored on memory devices or transmitted over unsecured networks. Each researcher will have password-protected, online, cloud-based storage for sharing of study-related files. Study communications will be conducted via secured institutional email servers.

Survey data will include information on socio-demographic characteristics, household food security status, and adolescent food habits obtained using a standardized, food frequency questionnaire (FFQ). Survey data will be collected by the trained enumerators.

Venous blood samples will be collected by a trained phlebotomist; ~ 10 ml of blood will be collected from each participant using lithium heparinized vacutainers following an aseptic procedure and using a disposable syringe and needle. Vacutainers will be transported within 12 h of collection to the International Center for Diarrhoeal Disease Research, Bangladesh (ICDDRB), based in Dhaka. In the state-of-the-art facility laboratory, the serum will be separated and stored at a temperature of 2–8 °C and then analyzed. The blood collection per participant will take about 10 min. We propose to measure complete blood count (CBC), which includes the following: erythrocyte sedimentation rate (ESR), hemoglobin (Hb), hematocrit, packed cell volume (PCV), mean corpuscular hemoglobin (MCH); mean corpuscular volume (MCV) ; mean corpuscular hemoglobin concentration (MCHC), red blood cells (RBC), white blood cells (WBC) total count, differential count, platelet count; sFer, soluble transferrin receptor (sTfR), C-reactive protein (CRP), and blood ABO typing.

Five common measures of attentional (three attention tasks) and mnemonic functioning (two memory tasks) will be used to assess the use of cortical systems and circuits that have been documented to have some dependence on Fe status, based on the literature on either human or animal studies. These measures and tasks have been used extensively in human experiments, and have each been described previously [13, 14]. These computerized assessments will be used to measure attention and visual memory, as used in previous biofortification feeding trials in similar target populations [21–24]. Briefly, all tasks will be undertaken by pre-installed DMDX software (developed by the University of Arizona) and will include detailed instructions and practice tests [25]. This computer-based application will be administered by trained local research assistants.

The simple reaction time (SRT) task provides an estimate of the speed of the simplest possible behavioral response to a visual stimulus and requires a participant to press a button in response to the onset of the task stimulus [26]. The go/no-go (GNG) task provides an estimate of the efficiency of sustained attention and the speed of simple attentional capture without the need to filter information from any immediately competing stimuli [26]. It requires a participant to press a button in response to the presentation of an infrequent stimulus (presented on 20% of the trials) and to withhold a response to a frequent stimulus (presented on 80% of the trials). The attentional network task (ANT) is a modified flanker task that provides an estimate of the effectiveness of three distinct components of attention [27]. In each trial, a participant will be presented with either an informative or an uninformative cue and will be required to press a button to indicate whether an arrow in the center pointed to the left or right while attempting to disregard flanking elements. The cued recognition task (CRT) is a modified version of a classic, visual recognition, memory task that estimates the speed, accuracy, and efficiency of recognition based on short-duration visual memory [28, 29]. Participants will be presented with a set of pictures of common, easily named objects, after which an equal number of previously seen and new items will be presented, and participants will be required to indicate whether each item was in the original set or is new. The Sternberg memory search (SMS) task estimates the speed and accuracy with which immediate visual memory can be searched [30]. In each trial, participants will be first shown a set of 1, 3, or 6 simple graphical symbols to remember, then a test stimulus will be presented, and the participant will indicate whether she remembers the test stimulus from the previous set of symbols. It would take around 45 min per participant.

Data on how much cooked lentil was served to each adolescent girl and how much remained uneaten by each girl will be collected on a daily basis by the research assistants. This information will be used to determine the actual amount of cooked lentil consumed by the adolescents.

### Outcome measurement

#### Assessment of Fe status and anthropometrics

Fe status will be assessed by measuring hemoglobin, hematocrit (volume of red blood cells), sFer, (sTfR), CRP, and imputed total body Fe (TBI). The TBI (mg/kg) will be calculated using the ratio of sTfR to sFer [34].

Participants’ height, weight, waist, hip, and mid-upper arm circumference (MUAC) will be measured to determine change in growth [35–37]. Participants will remain bare-foot, wearing minimal clothing, and we will avoid carpets, sloping, rough, and uneven surfaces when performing anthropometric measurements. The Frankfurt horizontal plane will be ensured when measuring height, and participants will be requested to put their heels together. In addition, their backward curved body parts (buttocks and shoulder blades) and head will be placed against the plane. Participants will be requested to remove shoes and socks and stand still, facing forward, with the palms by their sides, for measurement of weight using a digital body weight bathroom scale. Waist circumference will be measured under the midline of the armpit and at the midline between the inferior part of the last rib and the top tip of the hip bone, using a constant tension tape. Hip circumference will be measured at the point of the maximum diameter of the buttocks using the same constant tension tape, which will also be used to measure the MUAC. First, participants will be requested to put their left arm at 90^°^ angles, and the midline will be marked between the proximal and distal points. The tape will be then wrapped around the point and measured ensuring that the tape will be neither too tight nor too loose. The measurement unit for all anthropometric data will be in centimeters (cm) except for weight, which will be captured in kilograms (kg). Participants’ mean body mass index (BMI) will be calculated using the BMI percentile calculator for children and teens [38].

Dietary intake will be assessed by a field research assistant at baseline and end of study using culturally appropriate FFQs.

#### Cognitive performance

Participants’ attention, and memory (number of correct responses and time (seconds and milliseconds)) will be measured using DMDX software.

### Statistical analysis

Baseline characteristics, i.e., all covariates, will be examined for group comparability to determine if randomization was successful. An intent-to-treat approach will be followed in the analyses of outcome data at the end of the intervention. Outcome variables such as sFer, and Hb will be set using different cutoffs from the National Micronutrients Status Survey 2011–12 [2]. Data will be analyzed with and without adjustment for baseline characteristics. Group means for changes in biochemical variables, anthropometric measures, and post-intervention morbidity rates will be compared using mixed models (with cluster as the random effect), with the respective baseline values serving as the primary covariate. After these preliminary analyses, key outcome variables (sFer and the cognitive performance of the adolescent girls) will be re-examined and additional covariates will be included in the model such as factors that differed by intervention arm, i.e., age, socio-economics, anthropometrics, food security, drinking safe water, hygiene practices etc.). Additional continuous outcome variables will also be examined using a generalized linear model (GLM), adjusted for confounding factors such as age, socio-economics, anthropometrics, food security, drinking safe water, hygiene practices, etc. Categorical variables will be compared using the chi-square test or logistic regression adjusted for possible confounding factors. With the assumption that data will be missing at random (MAR), multiple imputation or likelihood-based mixed models will be used for analysis, after complete case analysis and examination of missing data within and between treatment groups. All outcome variables will be examined for normality, and outliers will be identified. Data collected from adolescent girls suffering from severe Fe deficiency and/or acute infection will participate in all aspects of the study but results of supplementation in this group will be analyzed separately. All analyses will be performed using SPSS for Windows PAWS version 25. A *p* value <0.05 will be considered statistically significant for all main effects.

## Discussion

The study aims to measure the effectiveness of consuming Fe-fortified lentils on the body Fe status and cognitive performance in adolescent girls. Cooked Fe-fortified lentil (dal) is the only intervention considered in this study. To measure its effectiveness, we controlled other covariates that may have influence over the study outcomes: we are providing non-Fe-fortified lentils; cooked rice is being provided to increase compliance (based on the previous pilot study findings); and participants are being dewormed by albendazole at the time of feeding trial [31–33].

Adolescent girls are prone to Fe deficiency, particularly in resource-poor settings, due to a variety of factors. Although this study is limiting participation to adolescent girls, we will serve the Fe-fortified lentil to all children and teens who are participants in the BRAC Adolescent Club during the intervention period for the purposes of equity. Adolescents who do not meet the inclusion criteria, however, will not be included in the analyses. Spill-over effect is less likely to occur as the study does not provide knowledge or any other information that may affect the study outcomes, which are biological in nature.

Another aspect that may affect our outcomes is that as the study proposes to serve the same recipe for ~ 4 months, it is possible that consumption of a single recipe daily for this length of time could lead to boredom among participants and may reduce consumption or may result in drop-outs after several weeks. We predicted this situation would occur and attempted to address it in our pilot study in 2016–2017, which ran for 3 months and tested two different recipes (a thick and a thin preparation) given in three different amounts, using local ingredients and cooking procedures. This study came up with a single recipe that was found to be most acceptable to the participants (BRAC Adolescent Clubs in nearby Gazipur district), and we did not have drop-outs or reduced consumption due to palatability. Furthermore, it is important for the study to maintain its rigor and standardize the intervention recipe in order to capture the true effect of the Fe-fortified lentils compared to normal lentils and/or usual intake. So, the probability of any fluctuation in the feeding trial due to the recipe would equally affect each intervention arm, provided that we use the same recipe for the duration of the study among all arms. If we provided different recipes to increase the consumption, it is possible that the study participants would increase or decrease their consumption compared to the earlier recipe (even though the probability of fluctuation remains the same), and that could cause variation in the study outcome.

Quality control will be ensured by following multiple steps. For instance, direct training of enumerators, data quality control supervisors, and data managers will be carried out prior to the commencement of data collection to ensure equal adherence to the consent, assent, and questionnaire with the same degree of questioning and accurate understanding of the format. Furthermore, in this face-to-face interview approach using a close-ended questionnaire, response options will be strictly formatted. Field enumerators will be advised to cross-check the questionnaire among team members before sending it to the server using a cellular Internet data connection. Additional training will be provided to the data quality control supervisors at the field level, and the interview process will be spot-checked in order to uncover any mistakes in the data collection procedures.

All interviews and arthrometric measurements will be performed separately by experienced female interviewers in a private setting in the presence of a witness. Each arthrometric measurement will be taken three times to ensure consistency. Survey and daily data will be collected electronically by the Open Data Kit (ODK) app on an Android-based platform [39, 40]. This customizable mobile or tablet-based app can work both online and offline allowing the use of GPS tracking, setting the condition of the responses and enabling real-time data monitoring. An experienced data manager will be thoroughly checking the data received every day and will be providing feedback directly to the enumerators. Additionally, our data manager will be assessing the need for re-training the field staff.

The benefits of this study are threefold. First, the results of the study will be used to garner support and substantiate large-scale market expansion of Fe-fortified lentils in Bangladesh and in other countries where lentils are consumed as a staple. Second, the extensive BRAC country-wide program network has high potential for efficient marketing of high-Fe lentils throughout Bangladesh. Third, the results from this study will contribute to the knowledge on food-based approaches to enhancing the Fe status of adolescents worldwide in resource-poor settings.

Compared to the effect of Fe supplementation to improve Fe status, we expect a smaller effect size in this study. The study will require a greater number of samples to statistically detect the smaller differences between baseline, midline and endline, and between-groups status in mean sFer. The small increase in mean ferritin status has the potential to reduce the prevalence of Fe deficiency by shifting a certain proportion of the deficient population above the cutoff level (sFer > 12 μg/L). Considering the prevalence of Fe deficiency (sFer < 12 μg/L) of ~ 30% among female adolescents in rural areas, shifting the population mean sFer from 22.5 μg/L to 27.5 μg/L would reduce the prevalence to about 20%.

Three different platforms will be used for dissemination of the study results. The study findings will be presented at academic seminars at the University of Saskatchewan. Furthermore, the same results will be presented in a seminar that will be organized by the BRAC Research and Evaluation Division. A layperson’s summary of the results will be shared with BRAC for dissemination at the field level. The study findings will be presented at various conferences, such as the American Society for Nutrition (ASN), Canadian Society for Nutrition (CSN), University of Saskatchewan annual Life and Health Science Expo, and other nutrition and public health conferences. Manuscripts will be written based on the study findings and submitted to high-impact factor, peer-reviewed scientific journals Additional file 1.

### Trial status

Not yet recruiting.

## Additional file

Additional file 1: Standard protocol items: recommendation for interventional trials (SPIRIT) 2013 checklist: recommended items to address in a clinical trial protocol and related documents*. (DOCX 51 kb)

## Abbreviations

AC: Adolescent Clubs
CDC: Crop Development Centre
DDS: Dietary Diversity Score
DPS: Department of Plant Sciences
DSMB: Data Safety Monitoring Board
FFQs: Food frequency questionnaires
GoB: Government of Bangladesh
icddr,b: International Centre for Diarrhoeal Disease Research, Bangladesh
LSBE: Life-skill-based education
NaFeEDTA: Sodium iron ethylenediaminetetraacetic acid
sFer: Serum ferritin
sTfR: Soluble transferrin receptor
TBI: Total body Fe
UID: Unique identifier number
UofS: University of Saskatchewan
VAS: Visual analog scale
